# Some Medical Aspects of Tropical Colonisation

**Published:** 1891-02-21

**Authors:** 


### SOME MEDICAL ASPECTS OF TROPICAL COLONISATION.

Sir William Moore dealt with many questions of great and increasing interest in his paper read before the Epidemiological Society, on January 21st, 1891, entitled "Is Colonisation in Central Africa by Europeans Possible?" Many years ago the late Sir Henry Lawrence was initiating his philanthropic schemes for the establishment of schools for European children on the Indian hill ranges, and for the settlement of time-served soldiers as colonists on the Indian hill ranges. Sir W. Moore expressed his opinion that European colonisation could not succeed in India. And, as a matter of fact, during the 35 years which have elapsed there has been no European colonisation. Sir W. Moore was connected for a considerable time with one of the hill asylums for European children, founded by Sir Henry Lawrence, and a systematic inquiry into the future of the children leaving the school was instituted. The results were not altogether satisfactory, especially in the cases of females. Of the numerous Europeans who have settled in or near the great Indian stations none have been colonists. There is not a great grand-child of these people of pure European descent. It is the same with the Portuguese, once so powerful in India. And, if other tropical climates are referred to we do not find any in which the European race has established itself without admixture of native blood. Peru, Mexico, and several other localities were mentioned as exemplifying the above. Prolonged and continuous heat, especially when combined with moisture, produces more or less blood deterioration. The question is, can any locality be found under a tropical sun where Europeans can live, flourish, and reproduce their kind while, while obtaining their livelihood by tilling the ground? Such a locality has not been discovered in other parts of the world, and there is no reason to suppose that it will be found in equatorial or even semi-tropical Africa.

Africa lies almost entirely in the torrid zone, and is the hottest of all continents. From the shores of the Red Sea to 15 degrees south of the equator the mean temperature, so far as is known, is from 80 deg. to 85 deg., a condition which presents only in three other parts of the world. Then, especially in equatorial Africa, there are prolonged rainy seasons. Humidity and heat are the two conditions most unfavourable to Europeans. No one has yet proposed European settlements on the African coasts. It is the interior higher lands which have been pictured as the future sites of smiling European villages. No doubt the diminution of heat resulting from elevation is a considerable gain, but it must be recollected that elevation tells unfavourably on many people, exciting insomnia and hill diarrhœa. But to obtain a mean temperature such as that of London it would be necessary to ascend in Africa 10,000 feet. A more moderate mean temperature would still be favourable to the European, but so far as is known there are no elevated lands in Africa giving a mean temperature of, say, 65 deg. or thereabouts. There are mountains, as, for instance, Kilimanjara, which Dr. Hans Meyer has recently told us is 23,000 feet high. There is Mount Kenia, on the equator, about 6,000 feet high; there are mountains in Abyssinia 10,000 feet elevation, and there are the Livingstone Mountains, from 3,000 to 6,000 feet high. But the descriptions given of these hill regions show at once that they are not fitted for a race of European colonists. Unfortunately the lands most suited to cultivation in Africa are the lowlands bordering the great rivers and lakes, or, in other words, the parts of the country which are most unhealthy. We do not know very much about the diseases of Africa, but the following have been seen in one or other parts of the country: Cholera, beri-beri, dengue, endemic hæmaturia, guinea worm, leprosy, malarious fever, negro lethargy, oriental boil, plague, scurvy, small-pox, tænia, tropical dysentery and hepatic abscess, yaws, yellow fever, ulcers, and Stanley also mentions chest complaints and rheumatism. This is a formidable list for the European to face. There is one factor against Europeans flourishing in a tropical climate which would cease to be a factor if care were taken. But often Europeans proceed to a tropical climate who are totally unfitted for it either by constitution, temperament, hereditary tendency to certain maladies, or from the fact of already having suffered from certain maladies. No one should be allowed to go to a tropical climate without medical examination as to his or her fitness or otherwise.

An animated discussion ensued, in which the President, Dr. Ewart, Sir Joseph Fayrer, Dr. Willoughby, and Surgeon-General Murray took part. The prevalent idea was in favour of Sir William Moore's views, but at present we could only judge from analogy, and not from actual experience. Dr. Willoughby quoted Messrs. Thompson and Harrington, to the effect that a climate suitable for Europeans was to be found in British East Africa. But it must be recollected that British East Africa is an equatorial region, and, therefore, notwithstanding any elevation of moderate height, must still be a tropical region.

